# General Remarks on Aliments Produced by the Different Classes of the Animal Kingdom, and Their Influence on the Human Body

**Published:** 1800-03

**Authors:** J. J. Virey


### *General Remarks on Aliments produced by the different Claſſes of the Animal Kingdom, and their Influence on the Human Body.* By J. J. VIREY, &c.

[Concluded from our laſt Number, pp. 153—156.]

THE more we retreat from perfect animals, the nearer we approach thoſe which reſpire imperfectly, and from which we obtain coarſe and even dangerous aliment: thus we obſerve, that when an animal reſpires freely, it affords us a more nutritious albumen and jelly; we can in ſome degree aſcertain this nutritive property, and its reſpective ſalubrity, by the organs of reſpiration, and by thoſe of the circulation, which depend on the former. Hence the animals which are deprived

---

*without* thoſe anomalous aſpects, would not be worthy of publication. And after all, the whole of its credit is equivocal. The public will at leaſt concede, that if with experience equally extenſive, we ſtill find the reſults the ſame as originally ſtated, we have no right to reſign thoſe original pretenſions.

of theſe organs, are to us actual poiſons; and in proportion as we aſcend in the ſcale of animal compoſition, theſe poiſons diminiſh, and nutriment increaſes. We do not meet with any poiſonous quality in a natural ſtate in the fleſh of ſuch animals as have perfect organs of reſpiration, together with red and hot blood.*

If an ancient writer ſuppoſed that the affections of the human heart could be changed by aliment, it might alſo be poſſible to determine this influence on the phyſical ſtate of nations, with ſtill greater eaſe than their moral ſtate, which in a manner depends on the former. This field of knowledge being yet unexplored and neglected by travellers and naturaliſts, cannot here be ſketched without difficulty; it would not even form a ſubject of inquiry, if man was not naturally omnivorous. But let us content ourſelves with forming the contraſt between the two extremes, which alſo depend much on the nature of the country inhabited by man.

All the inhabitants of the hot and of the equatorial countries ought to be more herbaceous, and thoſe of the polar climates ſhould be more carnivorous; this may be eaſily accounted for. The carnivorous man would die under the line, by too great a quantity of nouriſhing aliment,† and the reverſe would cauſe the death of the phytophagyan under the pole. In the intermediate ſpace, we meet with others who intermix vegetable aliment with the fleſh of animals, according to the degrees of latitude in which they live. Vegetable food produces in the inhabitants near the equator a puſillanimous, irritable, feeble, mild, tranquil, ſpeculative, ſuperſtitious, avaricious, and careleſs ſtate of mind. All their energy is centered in the pleaſures of love, and the deluſions of imagination. The carnivorous, robuſt, and ferocious inhabitant of the polar regions, on the contrary, is brutal, without moderation or religion, courageous even to raſhneſs; free, ſprightly, and inconſtant, his judgment is as dull‡ as his ſenſes are coarſe;

* The venom which proceeds from the bite of ſerpents, is only a ſort of very powerful digeſtive liquor, forming their gaſtric juice, and the action of which is ſlow. Several animal humours may degenerate and become venomous by the paſſions and by diſeaſes; a circumſtance which apparently indicates a ſort of vitality.

† The inflammatory diſeaſe to which all Europeans are ſubject who go to the Indies, ariſes from no other cauſe. It partly reduces their plethoric ſtate, which is ſo neceſſary in cold climates.

‡ Cit. Virey appears, in this inſtance, to diſplay that fondneſs for *generalizing* ſuperficial obſervations, and that propenſity for drawing haſty concluſions, which in a peculiar manner characterizes the bulk of his countrymen. When coarſe; in him every thing expreſſes durability; his energy is a kind of furor; and his voluptuouſneſs, exceſs. The former is expoſed to the moſt abject ſlavery, the latter becomes a moſt intolerable tyrant. If both are conſidered in a political point of view, what an immenſe ſcene is open for our inveſtigation! How can all this be deſcribed? What an immenſe detail, eſpecially when in this inquiry, thoſe nations be included which fill the intermediate ſpace between thoſe extremes. The immortal Monteſquieu has well conceived it. I here lay down my pen.

Non mihi, ſi linguæ centum ſint, oraque centum,

Ferrea vox. VIRGIL, ÆNEID. vi.

Let us only examine ſome differences reſulting from the uſe of each claſs of animals. Though there are no nations who confine themſelves to one particular kind of food, nevertheleſs the natural production of the country may oblige them principally to ſubſiſt upon it.

The nations who live on the fleſh of quadrupeds, and even on that of birds, are thoſe in particular who inhabit the northern countries of Europe,—ſuch as the Swedes, the Danes, the Ruſſians, the Germans, and the Engliſh; and in northern Aſia, the greater part of the hordes of Tartars and Kalmucs. Their behaviour exhibits a certain roughneſs or haughtineſs, an energy, and even firmneſs of mind, more or leſs characteriſtic. This ſort of firmneſs frequently changes, even into an invincible obſtinacy among the nations ſubſiſting by the chace, and the ſavages of North America. The diſeaſes of theſe nations, eſpecially, are of a plethoric and inflammatory nature, and depend chiefly on the ſanguiferous ſyſtem.

There are only a few miſerable individuals on earth, who are contented with feeding on reptiles; but as they do not confine themſelves to theſe alone, we can form no certain concluſions. It has however been remarked, that they are ſubject to cutaneous herpetic affections, and ſometimes even to gonorrhœa, or rather to habitual blennorrhagias. All this is obſervable in a greater or leſs degree among the black inhabitants of Thebes, of the mountains of Paleſtine, among the wretched Cophtes, the Druſes, &c.

When attributing this unfavourable character to the northern nations, he ſeems to have forgotten that they have produced a modern Semiramis; that their merchants and political ſpeculators far excel in cunning and intrigue, the revolutionary heroes of the ſouth; and that they alſo boaſt of philoſophers of the firſt eminence.

The Ichthyophagous nations are very numerous, and cover the ſea-ſhores in multitudes, ſo that they would ſoon deſtroy the fiſh, if their fecundity was not inexhauſtible. They contract, it may ſaid, the nature of palmipedous birds, who enjoy the ſame kind of life as themſelves; they are very voracious, and for this reaſon are very prolific. They are intrepid ſailors, but puſillanimous ſoldiers; they are alſo groſs, barbarous, and ſtupid. Their diſeaſes, which are chiefly endemic, attack the lymphatic ſyſtem, and the organ of the ſkin. The latter affections act as a conſiderable ſtimulus on the parts of generation; and this, united to the abundance of food, muſt conſiderably increaſe population.

With reſpect to the Entomophagous hordes, their diſpoſition appears as weak and contracted as their food is wretched; they are, and always will remain, ſtupid and barbarous. This ſtimulating and acrid aliment, renders them thin and dry: the diſeaſes incident to them, attack the organs of contractility, and produce many ſpaſmodic affections. It is aſſerted, that they are ſubject to phthiriaſis; in other reſpects, theſe nations which chiefly exiſt in Africa, are not ſatisfied with inſects alone, and the drying heat of the climate has alſo a powerful influence on their conſtitutions.

It is difficult to compute thoſe ſcattered tribes, who procure with great efforts a pitiful ſubſiſtence on the ſandy ſhores, by ſearching for ſhell-fiſh.

Of this deſcription are ſome of the inhabitants of the iſles in the South Sea, and in New Guinea; they are weak, timid, and perfectly ſtupid. They are alſo treacherous; for as they cannot drive away by main force the ſtrangers who famiſh them by viſiting their iſlands, they endeavour to attain their aim by ſtratagem.

There are not a ſufficient number of obſervations made on their phyſical condition, to enable us to draw any practical reſult.

It is worthy of notice, that all the maladies which ariſe from the nature of their food, extend their action particularly to the ſkin; a circumſtance which proves the ſtrong ſympathy by which it is connected with the inteſtinal canal.

All that I have ſaid reſpecting animal food, as uſed by various nations, can only be conſidered as an attempt towards an analyſis of this intereſting ſubject, which ſtill remains ſo obſcure and thorny, that it is difficult at firſt to diſcover the true path.

However, there can be no very great deviation, while we remain at the veſtibule of uſeful inquiry. We are not yet enabled to take a comprehenſive and ſatisfactory view of the whole, as as the present sources of information on this subject are united in many respects to Natural History, Physics, Medicine, and Ethics.

